# Demographics, social position, dental status and oral health-related quality of life in community-dwelling older adults

**DOI:** 10.1007/s11136-015-1209-y

**Published:** 2015-12-26

**Authors:** Maria Augusta Bessa Rebelo, Evangeline Maria Cardoso, Peter G. Robinson, Mario Vianna Vettore

**Affiliations:** School of Dentistry, Federal University of Amazonas, Rua Rio Itannana, 111, Bairro Nossa Sra das Gracas, Manaus, AM CEP: 69.053-040 Brazil; Academic Unit of Dental Public Health, School of Clinical Dentistry, University of Sheffield, 19 Claremont Crescent, Sheffield, S10 5SX UK; School of Health Sciences, State University of Amazonas, Av. Djalma Batista, 2470, Chapada, Manaus, AM CEP: 69050-10 Brazil

**Keywords:** Oral health, Quality of life, Elderly people, Socioeconomic status, Structural equation modelling

## Abstract

**Purpose:**

To identify demographic, socioeconomic and dental clinical predictors of oral health-related quality of life (OHRQoL) in elderly people.

**Methods:**

Cross-sectional study involving 613 elderly people aged 65–74 years in Manaus, Brazil. Interviews and oral examinations were carried out to collect demographic characteristics (age and sex) and socioeconomic data (income and education), dental clinical measures (DMFT, need of upper and lower dentures) and OHRQoL (GOHAI questionnaire). Structural equation modelling was used to estimate direct and indirect pathways between the variables.

**Results:**

Being older predicted lower schooling but higher income. Higher income was linked to better dental status, which was linked to better OHRQoL. There were also indirect pathways. Age and education were linked to OHRQoL, mediated by clinical dental status. Income was associated with dental clinical status via education, and income predicted OHRQoL via education and clinical measures.

**Conclusion:**

Our findings elucidate the complex pathways between individual, environmental factors and clinical factors that may determine OHRQoL and support the application of public health approaches to improve oral health in older people.

## Introduction

The oral health of older people is increasingly important. First of all, the global demographic transition means that the number of older people is growing in most societies. There has also been a concomitant oral health transition, with older people retaining their teeth for longer, so increasing their dental treatment needs [1, 2]. In addition, the effects of risk factors and oral diseases through the lifespan are cumulative, so threatening their overall health, quality of life and well-being [1].

The oral health of older people has been traditionally assessed using normative clinical measures (e.g. tooth loss) in epidemiology. However, this traditional approach ignores the social, emotional and functioning aspects of oral health [3]. Thus, subjective indicators have been adopted to assess the extent to which oral health problems impact on physical functioning and psychological and social well-being. One such measure, oral health-related quality of life (OHRQoL) represents the subjective experience of symptoms related to oral conditions that impact on psychosocial well-being. OHRQoL can be used as an outcome measure to assess the determinants of oral health and to evaluate the effectiveness of health promotion and dental treatment [5].

Demographic and socioeconomic characteristics as well as dental clinical status influence OHRQoL in older adults [6, 7]. A clear gradient between social position and OHRQoL in older adults has been reported; for example, lower levels of education are related to greater impact from oral conditions on everyday life [6–8]. Other sociodemographic factors, such as transport constraints, race, income and education, have also been related to OHRQoL [9, 10].

The association between clinical indicators and the OHRQoL of older people has not been fully elucidated. Having more teeth and more occluding pairs of teeth predicts better OHRQoL [8]. In addition, a systematic review concluded that the distribution of tooth loss affected OHRQoL [11]. However, there are inconsistent findings with respect to the effects of decayed teeth and on OHRQoL, with some studies finding a correlation [12–15], which is absent in other findings [16–18].

**Fig. 1:**
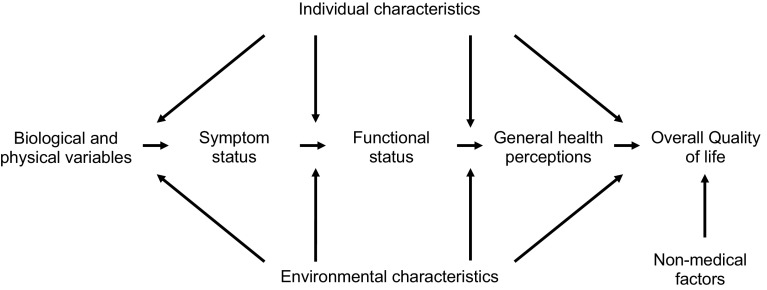
The Wilson and Cleary model linking clinical variables with quality of life

Few of the studies that have explored predictors of OHRQoL in older adults have been guided by a conceptual model to assess the simultaneous roles of demographic, socioeconomic and clinical factors [19]. The Wilson and Cleary model [20] organises the different types of health outcomes on five levels (Fig. 1). The *biological and physiological factors* consider biological and clinical status. *Symptoms measures* individual’s perception of physical, emotional and cognitive status. *Functional status* refers to the ability to perform defined tasks. *General health perceptions* are subjective ratings integrating all of the health concepts, and *overall quality of life* includes peoples’ subjective well-being through general measures of satisfaction. The causal links between adjacent and non-adjacent levels in the model may be influenced by individual and environmental factors.

Structural equation modelling (SEM) is well suited to analysing relationships within such a complex model as it can identify direct and indirect effects. Relationships between multiple independent variables and the outcome are analysed simultaneously to determine whether a pre-specified theoretical model is supported by empirical data [21].

Previous research has employed SEM to investigate clinical, psychological and social determinants of OHRQoL in patients with xerostomia, in children, in adolescents and in adults of working age in India [22–25]. However, there is a lack of studies using an explicit explanatory model to assess the relationships between social inequalities and oral health outcomes among older people [26]. A previous study supported the use of the Wilson and Cleary model with older people as symptom status determined daily functional status, which in turn influenced global oral health perceptions [19]. However, those findings were limited to edentulous older people receiving dentures. Moreover, demographic and socioeconomic characteristics were not investigated. No study has assessed the simultaneous roles of demographic and socioeconomic characteristics and dental clinical measures on OHRQoL in a representative sample of older adults using an explicit model.

**Fig. 2:**
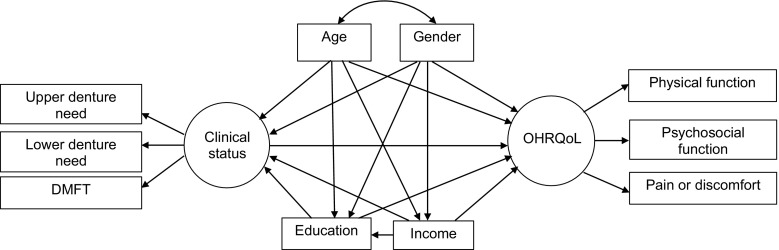
Full theoretical model on the relationships between demographic and socioeconomic characteristics, dental clinical measures and OHRQoL in older people according to Wilson and Cleary conceptual model

The present study aims to identify possible demographic (age and gender), socioeconomic (education and income) and dental clinical (dental caries and need for dentures) predictors of OHRQoL (GOHAI: physical function, psychosocial function and pain or discomfort) in older people, using the Wilson and Cleary conceptual model [20] (Fig. 2). The specific objective was to assess the extent to which social and demographic factors intervene in the effect of the clinical state of the mouth on OHRQoL.

## Materials and methods

### Study design and sampling procedures

A home-based cross-sectional study was carried out in the city of Manaus, state of Amazonas, Brazil, to obtain primary data regarding sociodemographic data, oral health clinical measures and oral health and quality of life from older people aged 65–74 years.

A stratified random clustered sample was drawn to obtain a representative sample of the 27,853 older residents living in Manaus, distributed according to the administrative regions of the city: Centre-South, Midwest, East, North, West and South.

The sample was obtained from census tracts, based on the proportion of the local population within each stratum. Full information regarding the sampling procedures was published elsewhere [28].

Exclusion criteria were people whose health conditions prevented dental examination and those who did not achieve the minimum score of a cognitive test, determined by the Verbal Fluency Test [29]. The project was approved by the Ethics Committee of the Federal University of Amazonas (Protocol No. 0234.0.115.000-07).

A sample size of 613 people was selected to lend a power 80 % detected a minimum effect size of 0.05 in an SEM at 5 % of significance (*α* = 0.05) directed towards hypothesis testing for complex models with 2 latent variables and 4 observed variables [27]. Initially, 810 older people were invited, of whom 44 declined to participate, 12 did not reach the minimum score of the cognitive test and 84 could not tolerate dental examinations due to poor health. Of the 667 selected, a further 54 were excluded because of incomplete data, resulting in a final sample of 613 older people.

### Measures

The measures were selected to operationalise a modified Wilson and Cleary [20] framework (Fig. 2).

### Clinical status

Clinical status was a latent variable created from three indicators: upper denture need, lower denture need and dental caries and treatment experience, collected in accordance with the criteria proposed by the World Health Organization [30]. Dental caries and treatment experience was assessed using the decayed, missing and filled teeth index (DMFT). Each sound tooth was coded as “0”, whilst each decayed, missing or filled tooth was coded as “1”. Then, the codes were summed to obtain a final DMFT score for each person.

Upper or lower denture need recorded the need for new full dentures in participants who had no natural teeth in the upper or lower jaws. Participants whose existing dentures required replacement on grounds of retention, stability, fixation or aesthetics were also deemed to have denture needs. Denture need was registered as “0 = no denture need” or “1 = denture need” for each dental arch with a final score ranging from 0 to 2.

The clinical measures variable was obtained as the sum of the DMFT and denture needs scores with higher scores denoting worse oral health status. The number of natural teeth was also recorded.

The oral examinations were conducted by a single and previously calibrated examiner using artificial head light, oral plain mirror No. 5 (Duflex^®^) and CPI periodontal probe (Stainless^®^) at the participants’ residences and in accordance with the biosafety rules. Test–retest reliability of the clinical measurements was determined in twenty older people attending a public community centre over 7 days. Kappa coefficient was 0.97 and 1 for DMFT and need for dentures, respectively.

### Oral health-related quality of life

Oral health-related quality of life was a latent variable measured by the 12-item Geriatric Oral Health Assessment Index (GOHAI) [5]. Originally developed for older people, GOHAI assesses the impact of oral health conditions on everyday life over a 3-month reference period on 3 dimensions of: (1) physical function, including eating, speech and swallowing; (2) psychosocial functions, including concerns about oral health, self-image and avoidance of social contacts because of oral health problems; and (3) pain or discomfort, including the use of medication to reduce pain or discomfort related to oral health problems [5, 31]. Participants respond on three-point Likert scale as follows: “Always” [1], “Sometimes” [2] or “Never” [3]. The scores for three items [3, 5, 7] were reversed. The item scores were summed to obtain a total GOHAI score, with a possible range from 12 to 36. Higher total scores denoted lower impact from oral conditions and thus better oral health-related quality of life. The internal reliability of GOHAI scale within these data was good (Cronbach’s alpha = 0.75).

The observed variables included age (complete years), gender and socioeconomic characteristics, including education (years of schooling) and income (monthly income per capita).

### Statistical analysis

Analysis was conducted in 3 phases. A preliminary analysis described the distribution of all variables. Edentulous (missing all natural teeth) and dentate (presence of at least one natural tooth) participants were compared for categorical and continuous variables using Chi-square and Mann–Whitney tests, respectively. Second, hypothesised measurement models were tested in confirmatory factor analysis (CFA) to confirm the associations between the latent variables and their observed measures. Finally, structural equation models examined the direct and indirect relationships between the observed and latent variables within the Wilson and Cleary model. We predicted a priori that environment (education and income) and individual (age and gender) characteristics would predict the latent clinical and OHRQoL variables. Specifically, we hypothesised that low schooling and low income would directly predict poor clinical status and poor OHRQoL. In addition, greater age and female gender were hypothesised to predict poor clinical status and OHRQoL. Indirect effects of schooling and income on OHRQoL via clinical measures were also hypothesised.

The total effect, which represents the sum of the direct link from one variable to another and the indirect effects where the link is mediated by other variables (e.g. income to OHRQoL mediated by clinical measures), was estimated by AMOS. Total indirect effects represent the sum of one or more specific paths. The bias-corrected bootstrap CI was used to assess mediation by analysing the statistical significance of indirect effects [32]. After estimating the full model, we removed non-significant direct paths to generate a statistically parsimonious model, which was re-estimated and compared to the full model with the Chi-square test.

Maximum likelihood estimation and bootstrapping were estimated using AMOS 22.0. Nine hundred bootstrap samples were re-sampled from the original data set to derive less biased standard errors and 95 % confidence interval (CI) bootstrap percentiles. Chi-square test statistic was used to assess the adequacy of overall model fit. We also used the root-mean squared error of approximation (RMSEA) with 90 % CI and goodness of fit (GFI), and the comparative fit indices (CFI). The threshold for a good model fit was RMSEA ≤ 0.06 and GFI and CFI values ≥0.95 [33].

## Results

The final sample consisted of 613 older people (69.5 % women, mean age 69.27 years (SD = 3.01) and 48.4 % dentate). The mean DMFT was 29.24 (SD = 3.96). The mean years of schooling and personal monthly income were 4.58 years (SD = 4.27) and US $364.67 (SD = 469.48), respectively. One Brazilian minimal wage was U$ 197.28 in the period of study (Table 1).

**Table 1:** Demographic and socioeconomic characteristics, clinical measures and OHRQoL (GOHAI); comparisons between edentulous and dentate participants

	Total *N* = 613	Edentulous *N* = 316	Dentate *N* = 297	*P* value
Demographic data				
Age, mean (SD)^a^	69.27 ± 3.00	69.55 ± 3.04	68.96 ± 2.93	0.087
Gender *n* (%)^b^				<0.001
Female	426 (69.5)	242 (76.6)	184 (62.8)	
Male	187 (30.5)	74 (23.4)	113 (38.3)	
Socioeconomic characteristics				
Schooling, mean (SD)^a^	4.58 ± 4.27	3.68 ± 3.38	5.55 ± 4.88	<0.001
Per capita income (U$), mean (SD)^a^	364.67 ± 469.48	355.45 ± 521.13	374.48 ± 408.07	0.097
Clinical variables				
DMFT, mean (SD)^a^	29.24 ± 3.96	32.00 ± 0.00	26.31 ± 3.97	<0.001
Upper denture need, *n* (%)^b^	265 (43.2)	165 (52.7)	100 (33.67)	<0.001
Lower denture need, *n* (%)^b^	216 (35.2)	187 (59.8)	29 (9.76)	<0.001
Number of teeth, mean (SD)^a^	3.88 (5.36)	0.0 (0.0)	8.01 (5.13)	<0.001
Functioning				
GOHAI				
Total, mean (SD)^a^	33.90 ± 2.70	33.90 ± 2.86	33.91 ± 2.53	0.362
Physical function, mean (SD)^a^	11.30 ± 1.18	11.20 ± 1.32	11.39 ± 1.03	0.122
Psychosocial function, mean (SD)^a^	14.27 ± 1.26	14.35 ± 1.30	14.18 ± 1.22	0.003
Pain or discomfort mean (SD)^a^	8.34 ± 1.00	8.34 ± 1.03	8.33 ± 0.96	0.323

^a^Mann–Whitney test

^b^Chi-square test

**Fig. 3:**
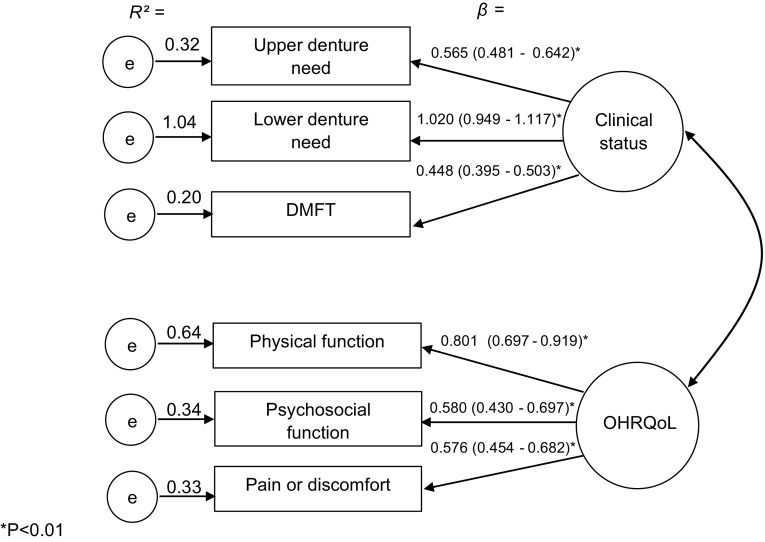
Confirmatory factor analysis of the 2-factor 6 items (measurement model) obtained through bootstrap item loadings (SE/BC 95 % CI)

Confirmatory factor analysis (CFA) supported the presence of (Fig. 3) individual latent factors (dental clinical measures and OHRQoL). The item loadings confirming the presence of “dental clinical measures” were as follows: upper denture needs (*β* = 0.565), lower denture needs (*β* = 1.020) and DMFT (*β* = 0.448). The loadings confirming OHRQoL were as follows: physical function (*β* = 0.801), psychosocial function (*β* = 0.580) and pain or discomfort (*β* = 0.576). Of these, the highest *R*^2^ was 1.04 (lower denture needs) followed by physical function (0.64), psychosocial function (0.34), pain or discomfort (0.33), upper denture needs (0.32) and DMFT (0.20).

SEM supported the hypothesised model with the values: SRMR = 0.042, RMSEA = 0.06, GFI = 0.973, CFI = 0.933. The regression weights showed that gender did not correlate with any variables. Thus, this variable and non-significant direct paths were removed to enhance statistical parsimony. The values of both models are presented in Table 2.

**Table 2:** Fit indices for the confirmatory factor analysis of full, measurement and parsimonious models

Model	*χ* ^2^ (*df*) (*P*)	GFI	CFI	SRMR	RMSEA
Full	3.552	0.973	0.933	0.042	0.06
Measurement model	1.849	0.992	0.990	0.031	0.037
Parsimonious	2.231	0.982	0.966	0.036	0.047

Model full = theoretical model adapted from Wilson and Cleary conceptual model. Measurement model = confirmatory factor analysis to between latent variables (clinical measures and OHRQoL). Parsimonious model = associations between clinical measures, age, education, income and OHRQoL with multiple direct and indirect effects model with pathways between all adjacent and non-adjacent levels. χ^2^ (*df*) (*P*) = Chi-square and degrees of freedom; *GFI g*oodness-of-fit statistics, *CFI* comparative fit index, *SRMR* standardised root-mean-squared residual, *RMSEA* root-mean-square error of approximation

**Fig. 4:**
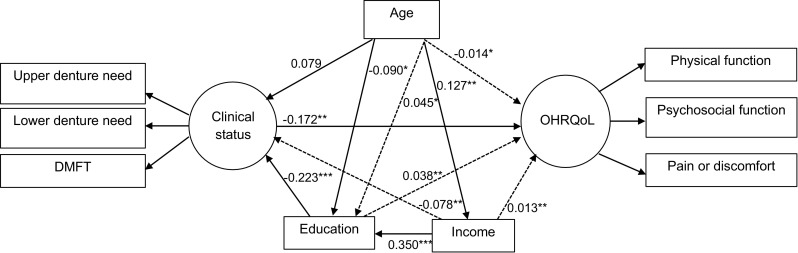
Parsimonious model of associations between clinical measures, age, education, income and OHRQoL. The variable “gender” was removed from this model as it was not statistically correlated with any variables. *<0.05; **P < 0.01; ***P < 0.001. *Solid lines* direct effects, *dashed lines* indirect effects [the total indirect effects were calculated as follows (all figures are standardised beta coefficients): (1) age to OHRQoL: age—clinical measures—OHRQoL = 0.079 × −0.172 = −0.014; (2) education to OHRQoL: education—clinical measures—OHRQoL = −0.223 × −0.172 = 0.038; (3) income to clinical measures: income—education—clinical measures = 0.350 × −0.223 = −0.078; (4) income to OHRQoL: income—education—clinical measures—OHRQoL = 0.350 × −0.223 × −0.172 = 0.013]

The parsimonious model shows good fit, meeting all our a priori criteria (Table 2). The direct paths in this model are summarised in Fig. 4. Being older was linked to lower schooling (*β* = −0.090) and higher personal monthly income (*β* = 0.127). Lower personal monthly income was linked to lower education (*β* = 0.350). The latter was linked to poor dental clinical measures (*β* = −0.223). Thus, low education and personal monthly income were linked to worse dental clinical measures, which in turn was linked to worse OHRQoL (*β* = −0.172). As shown in Fig. 4 and in line with our hypothesis, clinical factors predicted OHRQoL; having denture needs and higher DMFT predicted more severe impacts from oral conditions on everyday life (Table 3).

**Table 3:** Direct effects of the parsimonious structural equation model on the relationships between demographic and socioeconomic characteristics, clinical measures and OHRQoL

	*β*	Bootstrap SE	Bias-corrected 95 % CI
Age—income	0.127	0.039	0.054/0.206**
Age—education	−0.90	0.036	−0.157/−0.013*
Age—clinical measures	0.079	0.042	−0.002/0.165
Income—education	0.002	0.042	0.264/0.433**
Education—clinical measures	−0.223	0.043	−0.308/−0.133**
Clinical measures—OHRQoL	−0.172	0.057	−0.273/−0.053**

* *P* < 0.05; ** *P* < 0.01

*β* bootstrapped standardised estimate, *SE* standard error, *CI* confidence interval

## Discussion

The present study was a comprehensive investigation of individual socioeconomic determinants and dental clinical predictors of OHRQoL in older people. It was based on a explicit hypothesised theoretical model using SEM, which is considered the state-of-art approach to evaluate these relationships. The use of a representative and random sample suggests that our findings may be applicable to other cities with similar demographic and socioeconomic characteristics. Poor socioeconomic characteristics directly predicted worse dental clinical status and consequently worse OHRQoL. These data reveal how socioeconomic inequalities in OHRQoL may arise.

Different direct and indirect links were found. Dental clinical status was the only direct predictor of OHRQoL. Low education and low income predicted poor OHRQoL mediated through dental clinical status. Thus, the data are compatible with direct effects of clinical status on OHRQoL. Although seen in some previous research, confirmation of this relationship is important because other studies have not assessed clinical oral status [19, 34]. In addition, the variations in clinical status (i.e. both edentulous and dentate older adults) in our sample were greater than in previous studies that have been conducted in populations with relatively homogeneous oral status, such as children, adults with low caries levels and treatment experience and edentulous older adults [19, 23, 25]. Age showed contrasting relationships with socioeconomic indicators as it was inversely associated with education but positively associated with income.

Whilst our findings give new insights into the determinants of OHRQoL, they triangulate with the existing knowledge in this field. Our results are in agreement with previous studies showing the relationship between socioeconomic indicators and poor oral health in older people [7, 8, 31]. However, we have simultaneously shown the importance and interrelationship of socioeconomic and clinical indicators on OHRQoL. Age and income directly predicted clinical status and age, education and income indirectly predicted OHRQoL. For instance, lower income predicted poor OHRQoL mediated by education and dental clinical measures, and low education affected poor OHRQoL via dental clinical measures. These findings support and partly explain the socioeconomic inequalities on oral health [35]. For instance, whilst our findings on the relationship between socioeconomic factors and OHRQoL are supported by previous research [7, 36–38], other studies have not detected oral health inequalities among older people [39] or edentate older UK adults [40].

The potential explanations for these differences are the use of different instruments to assess OHRQoL, the variations in social indicators and the analytical approach to test the relationships. The present study benefits from the advantages of SEM by simultaneously analysing complex direct and indirect relationships within a previously stated causal model [21].

The use of a latent variable to summarise dental status (caries experience and denture needs) may explain its strong relationship with OHRQoL. Our results are compatible with previous findings of the associations between denture wearing and OHRQoL in older adults [41–44]. However, the incorporation of DMFT, of which missing teeth was main component (data not shown), may have highlighted the strong association between dental status and OHRQoL and suggests that the selection of clinical indicators warrants greater attention in dental epidemiology.

The GFI of the parsimonious model supports the application of the Wilson and Cleary framework to research with older people, corroborating the findings of a previous study with edentulous older people [19]. The results also support the use of GOHAI as a valid and reliable measure of subjective oral health status for use in epidemiology [6, 7, 31].

Some limitations of this study must be considered. The data were analysed according to the hypothetical causal ordering of Wilson and Cleary. However, our cross-sectional design restricts interpretation of the causal processes underlying these oral health outcomes [45]. Nevertheless, clinical dental status, income and education are probably stable characteristics over time among older people, whereas GOHAI was assessed OHRQoL over the last 3 months. Symptom status, general health perceptions and overall quality of life are components of the Wilson and Cleary framework that were not included in this study.

## Conclusion

The present study is the first to provide evidence of the importance of dental status on OHRQoL as well as the mediating effect of clinical status on the link between socioeconomic characteristics and OHRQoL in older people using a theoretical model. Our findings suggest the need for public policies for attention to oral health of older and consequent improvement in quality of life.
